# Optimization design of cross border intelligent marketing management model based on multi layer perceptron-grey wolf optimization convolutional neural network

**DOI:** 10.1038/s41598-025-89534-8

**Published:** 2025-02-12

**Authors:** Zongping Lin, Jing Yang, Yabin Lian, Yanhong Chen, Zhonghui Huang, Kexin Ning

**Affiliations:** 1 School of Economics and Management, Quanzhou University of Information Engineering, Quanzhou, 362008 Fujian China; 2 School of Economics and Business, Xiamen City University, Xiamen, 361008 Fujian China; 3 Academy of Art & Design, Minnan Science and Technology College, Quanzhou, 362332 Fujian China; 4 Xiamen Meitu Mobile Technology Co., Ltd., Xiamen, 361008 Fujian China; 5 School of Arts and Media, Beijing Normal University, Beijing, 100091 China

**Keywords:** Multilayer perceptron, Cross border intelligent marketing system, Convolutional neural network, Social label, Item rating, Grey wolf optimization, Environmental sciences, Environmental social sciences

## Abstract

The cross-border intelligent marketing algorithm based on traditional linear models is relatively single in information feature extraction, making it difficult to effectively handle complex scenarios containing a large amount of implicit information in users and markets, resulting in poor personalized marketing effectiveness. To address this issue, this article proposes a cross-border intelligent marketing model that integrates rating information and user labels using a multi-layer perceptron grey wolf optimization and convolutional neural network (MLP-GWO-CNN). This model extracts implicit high-order information through nonlinear methods and can handle complex and sparse marketing data. Firstly, a dual path deep network structure was designed, in which one path was modeled using a multi-layer perceptron (MLP) to extract user interest features based on historical interaction ratings; Another path utilizes Convolutional Neural Networks (CNN) to extract semantic features from user label information and construct item feature representations. In response to the sensitivity of MLP algorithm to initial values and its tendency to fall into local optima, this paper uses GWO algorithm to optimize MLP. Next, the latent feature vectors generated by MLP and CNN are fused in the output layer to generate the final predictive marketing strategy last. Experiments were conducted using a real cross-border e-commerce dataset, and the results showed that compared with traditional recommendation algorithms, the MLP-GWO-CNN model proposed in this paper performs better in utilizing user tag information, effectively improving the accuracy and personalization of marketing recommendations. The accuracy of the model is over 89%, and the recall rate is over 90%.

## Introduction

With the accelerated development of globalization and the digital age, cross-border e-commerce has rapidly emerged as a bridge connecting global markets. In this process, traditional marketing methods face new challenges and cannot meet the diverse needs of users^1^. The widespread application of emerging technologies such as big data, artificial intelligence, and the Internet of Things has driven the rise of intelligent marketing. Intelligent marketing provides enterprises with more accurate market insights and personalized marketing strategies by deeply mining user behavior data and consumption preferences. Compared to traditional marketing methods, intelligent marketing relies on technologies such as big data analysis and machine learning to more accurately predict user needs, thereby helping enterprises improve marketing efficiency, increase user stickiness, and market competitiveness^2^.

In the cross-border e-commerce environment, intelligent marketing faces more complex challenges and opportunities. Due to the cultural backgrounds, consumer preferences, payment methods, logistics, and other factors involved in cross-border transactions in different countries and regions, traditional marketing models are difficult to adapt to this complex dynamic environment. Therefore, building an intelligent marketing system with high adaptability and scalability, which can flexibly adjust marketing strategies according to the personalized needs of cross-border users and market dynamics, has become the key to solving the problems of cross-border e-commerce^3,4^.

In the stage of intelligent marketing, enterprises can utilize massive user consumption behavior records and their attribute feature information, and analyze them through artificial intelligence algorithms such as machine learning and deep learning to gain insights into user preferences and needs. This data-driven intelligent analysis not only improves the efficiency of marketing activities, but also makes marketing strategies more targeted and effective. At the same time, the development of intelligent marketing technology also provides researchers with rich opportunities and challenges, especially in the fields of how to use new technologies for precision marketing and improve user experience^5^. The application scenarios of intelligent marketing mainly focus on two aspects. Firstly, focus on the consumption relationship between users and products, including news recommendations, video recommendations, etc., to recommend products that interest users. Secondly, marketing activities around users, goods and advertisers, such as Internet display ads, aim to accurately deliver ads to users who are most likely to click on or consume goods. Both of these applications fully demonstrate the advantages of data-driven intelligent marketing. Based on the above background, this article proposes a cross-border intelligent marketing system design based on Multi Layer Perceptron grey wolf optimization Neural Network (MLP-GWO-CNN). The system aims to fully utilize user rating information and social tag information, explore users’ potential interests and needs, and achieve precise marketing.

The contributions of this article are as follows:

1. This article proposes a user feature extraction module that combines MLP and CNN. MLP is used to process user rating data and mine potential preference features of users; CNN extracts semantic features of tags by analyzing the social label information annotated by users. The combination of the two can more comprehensively capture users’ personalized characteristics and enhance the system’s predictive ability.
2. This article proposes a fusion mechanism between user rating characteristics and social label characteristics, which combines user rating information with annotated label information to form a more comprehensive user feature matrix. In recommendation algorithms, the effective integration of user ratings and tag information can better reflect the true needs of users, thereby improving the overall performance of the recommendation system.
3. In this article, GWO is used to optimize the connection weights and biases of MLP, thereby avoiding the defect of MLP falling into local optima during training. This method utilizes the global search capability of GWO to effectively optimize the weight settings of multi-layer perceptrons, enabling them to find better global optimal solutions in a wider solution space.

## Related works

Cross border intelligent marketing utilizes artificial intelligence and data mining algorithms to significantly improve the efficiency of marketing processes and more accurately meet users’ personalized needs^6^. In recent years, cross-border intelligent marketing has received widespread attention and application worldwide, gradually evolving into an independent and critical research field. In intelligent marketing systems, recommendation systems are a core component that primarily focus on recommending suitable products, services, or content to users based on their historical behavior, interests, and preferences^7^. It provides personalized recommendations by analyzing user interaction data with products or services, thereby increasing user engagement and satisfaction. This section introduces the recommendation methods based on social labels and the fusion of convolutional neural networks.

The recommendation method based on social labels has received widespread attention in recent years. Many researchers not only use rating data, but also combine implicit data such as trust relationships and social labels to significantly improve recommendation effectiveness^8,9^.

Reference^10^ proposed a neighbor aware joint probability matrix factorization algorithm that combines social labels. This algorithm calculates the similarity between users and resources based on label similarity and selects neighbors. Then, construct the user resource rating matrix, user tag annotation matrix, and resource tag association matrix, use joint probability matrix decomposition to calculate the hidden feature vectors, and provide recommendations to users through parameter optimization. Reference^11^ proposed a social network recommendation method that combines social labels and trust relationships. This method integrates social trust relationships, item tagging information, and user item rating data through probabilistic factorization techniques, connecting the potential feature spaces of users and items from multiple dimensions, and achieving effective social recommendations through dimensionality reduction. Reference^12^ proposed a recommendation algorithm that combines social behavior and label behavior. It uses a gravity model to measure the attractiveness between user nodes to calculate the similarity of social behavior, and constructs a user preference model based on label information. The gravity formula is used to calculate the attractiveness between preferred objects, and finally combines the two information to generate recommendations. A label aware recommendation model based on attention mechanism is proposed in reference^13^ to address data sparsity, fuzziness, and redundancy. This model can effectively capture the diverse, label based potential features of users and items. Specifically, the model generates dense label based feature vectors for each user and item through embedding methods, which contain more implicit information and help improve recommendation accuracy. Although these social label based recommendation algorithms have improved recommendation quality compared to traditional algorithms, they still struggle to meet the needs of personalized recommendations when facing users and projects with a large amount of implicit information.

Convolutional neural network (CNN) is a type of feedforward neural network with deep structure, widely used in the field of deep learning^14,15^. The basic architecture of CNN includes input layer, convolutional layer, pooling layer, and output layer. The input layer is used to obtain input data, the convolutional layer is responsible for feature extraction, and the pooling layer extracts key features by reducing the number of parameter features to prevent model overfitting; The output layer is used to output the processed latent feature vectors. CNN is widely used in computer vision and has gradually been introduced into recommendation systems in recent years. By extracting implicit information features, CNN can analyze text content more deeply and improve recommendation accuracy^16,17^. Reference^18^ proposed a recommendation algorithm that combines dynamic collaborative filtering and deep learning, incorporating time factors into collaborative filtering and learning user and movie feature information through deep learning models to generate high-dimensional latent space user and movie feature latent vectors, which are then combined with dynamic collaborative filtering for recommendation. Reference^13^ proposed an improved CNN model LSPCNN for local similarity prediction to alleviate the problem of data sparsity in recommendation systems. This model iteratively adjusts the initial user item rating matrix to locally characterize user interest preferences, and then uses CNN to predict missing ratings, achieving personalized recommendations.

## Methodology

**Fig. 1:**
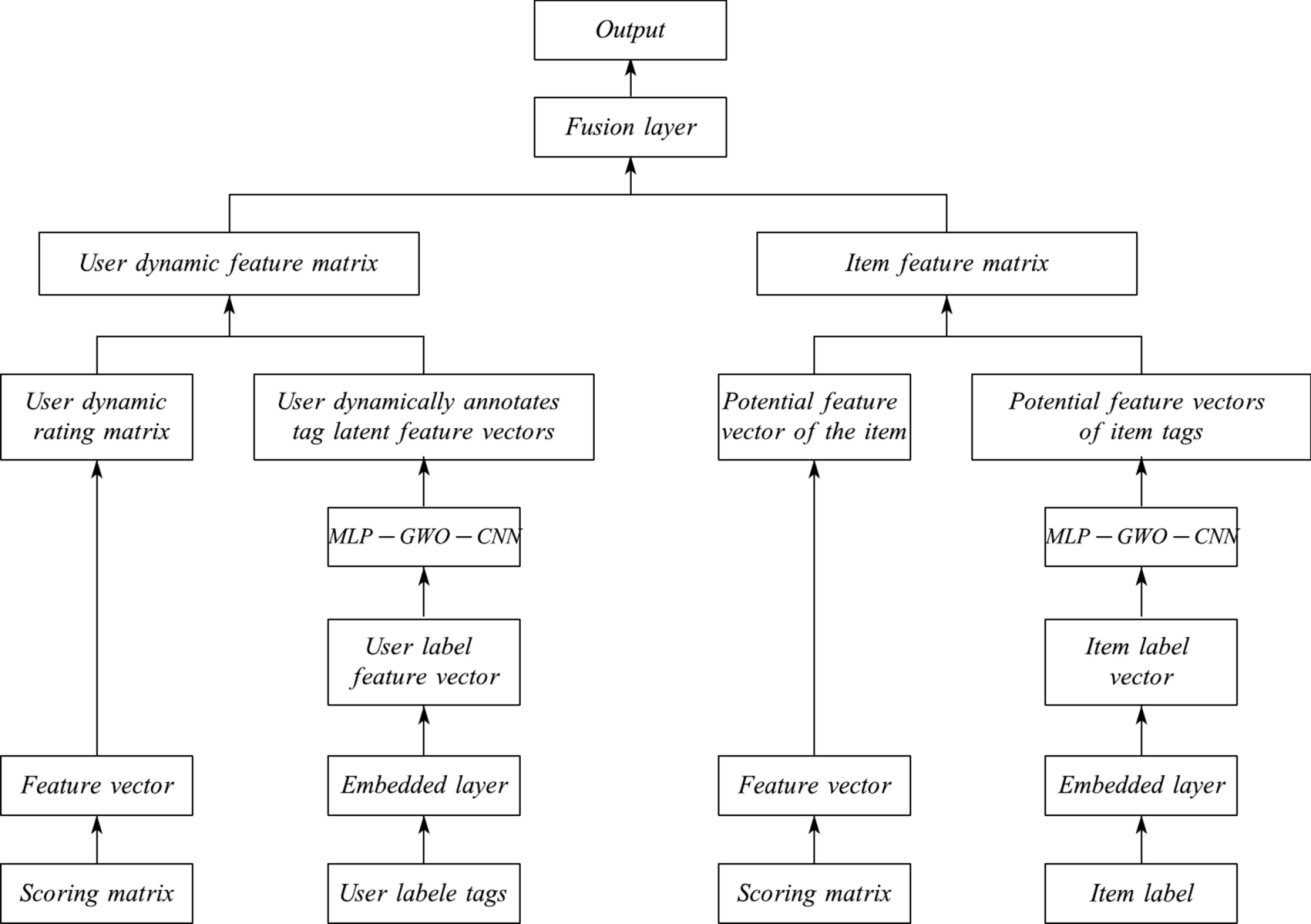
MLP-GWO-CNN model structure.

This article proposes a cross-border intelligent marketing model that integrates rating information and user labels using a multi-layer perceptron and convolutional neural network (MLP-GWO-CNN). Its structure is shown in Fig. 1. This model consists of four parallel neural networks. Firstly, two neural networks are used to extract the row and column vector information of the rating matrix from the perspectives of consumers and products, respectively, using a multi-layer perceptron to obtain the latent feature vectors of consumer ratings and product ratings. At the same time, the other two neural networks use convolutional neural networks to perform deep learning on the label information annotated by consumers and the product label information, respectively obtaining the latent feature vectors of consumer annotated labels and product labels. Next, the consumer rating features and annotated label features are fused to obtain the comprehensive feature matrix of the consumer; Similarly, the product rating features are fused with label features to generate a comprehensive feature matrix for the product. Finally, the comprehensive characteristics of consumers and products are input into the fusion layer for accurate prediction of cross-border intelligent marketing. This article mainly explains how to extract consumer features through multi-layer perceptrons and CNNs. The process of extracting product features is similar, so it will not be repeated.

### Multi-layer perceptron

The MLP is a forward structured artificial neural network that can map a set of input vectors to a set of output vectors. MLP consists of multiple node layers, each fully connected to the next layer. Except for the input node, each node is a neuron with a nonlinear activation function. MLP overcomes the limitations of single-layer perceptrons in processing linearly inseparable data and, through the ability of general function approximators, can approximate and abstract higher-level latent concepts. Due to the lack of prior knowledge about the distribution of latent concepts in MLP, it can identify complex patterns by extracting features from local regions. MLP, as a universal function approximator, follows the same training process as Convolutional Neural Networks (CNNs) by optimizing parameters through backpropagation. As a deep model, MLP has the ability to reuse features, improving parameter utilization and model output diversity. Therefore, this article proposes the Multi layer Perceptron Convolutional Layer (MPConv), which replaces traditional generalized linear models with multi-layer perceptrons to convolve input data and extract more abstract features from local regions of the image.

When using the Leaky ReLU activation function for feature extraction in the discriminator, the calculations performed by traditional linear MPConv are different. The traditional linear convolution layer convolves the input feature map with a linear filter, and the output result undergoes nonlinear transformation using the Leaky ReLU activation function. The expression is as follows:

1 $$f_{i,j,k} = \max (\alpha \omega_{k}^{{\text{T}}} x_{i,j} ,0)$$

2 $$\left\{ {\begin{array}{*{20}l} {f_{{i,j,k_{n} }}^{1} = \max (\alpha (\omega_{{k_{1} }}^{{1^{{\text{T}}} }} x_{i,j} + b_{{k_{1} }} ),0)} \hfill \\ \cdots \hfill \\ {f_{{i,j,k_{n} }}^{n} = \max (\alpha (\omega_{{k_{1} }}^{{n^{T} }} f_{i,j}^{n - 1} + b_{{k_{1} }} ),0)} \hfill \\ \end{array} } \right.$$

where, (i, j) represents the pixel index in the feature map, $x_{i,j}$ represents the input area centered around (i, j), k is used to index the channels of the feature map, n is the number of layers in the multilayer perceptron, $\alpha$ is the slope parameter of the negative value in the Leaky ReLU activation function, and f is the result of performing convolution operations on each layer data block and passing through the activation function.

### MLP based on grey wolf optimization algorithm

To address the issues of sensitivity to initial values, susceptibility to local optima, and slow convergence speed during MLP training, this paper adopts the GWO algorithm to optimize MLP. The GWO algorithm is a heuristic algorithm, and the most important step in training MLPs using heuristic algorithms as trainers is to choose an appropriate encoding strategy.

In this article, the matrix encoding strategy is chosen to represent the weights and biases of MLP as particle variables of the trainer during each iteration of the training process. Therefore, the variables of the trainer for MLP in this article are defined as:

3 $${\mathbf{V}} = \{ {\mathbf{W}},\theta \} = \{ w_{1,1} ,w_{1,2} , \ldots ,w_{n,n} ,\delta_{1} ,\delta_{2} , \ldots ,\delta_{h} \}$$

where n is the number of input nodes, $w_{ij}$ represents the connection weight between the i-th node and the j-th node, and $\delta$ represents the deviation of the j-th hidden node.

After defining the variables of the MLP trainer, it is necessary to define a fitness function for the MLP trainer, namely the GWO algorithm. The purpose of training MLP is to input training and testing samples into MLP, and use GWO algorithm to train and obtain appropriate connection weights and biases, so that MLP can achieve high classification accuracy, approximation and prediction accuracy for the target problem. Therefore, the mean square error (MSE) between the actual output value and the expected output value of MLP is used as an indicator to measure the performance of MLP. The smaller the MSE, the better the MLP performance, and vice versa.

#### Definition 1

MSE:Let $o_{i}^{k}$ represent the actual output value of the i-th output unit when the kth training sample is used as the input unit in MLP, and $d_{i}^{k}$ represent the expected output value of the i-th output unit when the kth training sample is used in MLP. The expression for MSE is

4 $${\text{MSE}} = \sum\limits_{i = 1}^{m} {\left( {o_{i}^{k} - d_{i}^{k} } \right)^{2} }$$

Obviously, Definition 1 is aimed at situations where there is only one training sample, however, actual research subjects often have more than one training sample. Assuming the research subject has s training samples, the $\overline{{{\text{MSE}}}}$ used to measure the performance of MPL becomes the average MSE of these s training samples, expressed as

5 $$\overline{{{\text{MSE}}}} = \frac{1}{s}\sum\limits_{k = 1}^{s} {\sum\limits_{i = 1}^{m} {\left( {o_{i}^{k} - d_{i}^{k} } \right)^{2} } }$$

where s is the number of training samples, and m is the number of outputs.

#### Definition 2

Trainer fitness function: If vector V is the variable of the MLP trainer and $\overline{{{\text{MSE}}}}$ is the average of MLP, then the fitness function expression of the MLP trainer is shown in Eq. (6)

6 $${\text{MinF}}(V) = \overline{{{\text{MSE}}}}$$

#### Definition 3

Trainer Classification Accuracy (CA): Classification accuracy, also known as “precision” or “accuracy”, is an important indicator for evaluating the performance of classification algorithms. Assuming that the set S to be classified has n attributes, the number of i-th attributes in the set S is denoted as $S_{i} (1 \le i \le n)$, and $T_{i}$ represents the number of correctly classified attributes i by the classifier, then the classification accuracy of the classifier for the set S to be classified is defined as

7 $${\text{CA}} = \left( {\sum\limits_{i = 1}^{n} {T_{i} } /\sum\limits_{i = 1}^{n} {S_{i} } } \right) \times 100$$

**Fig. 2:**
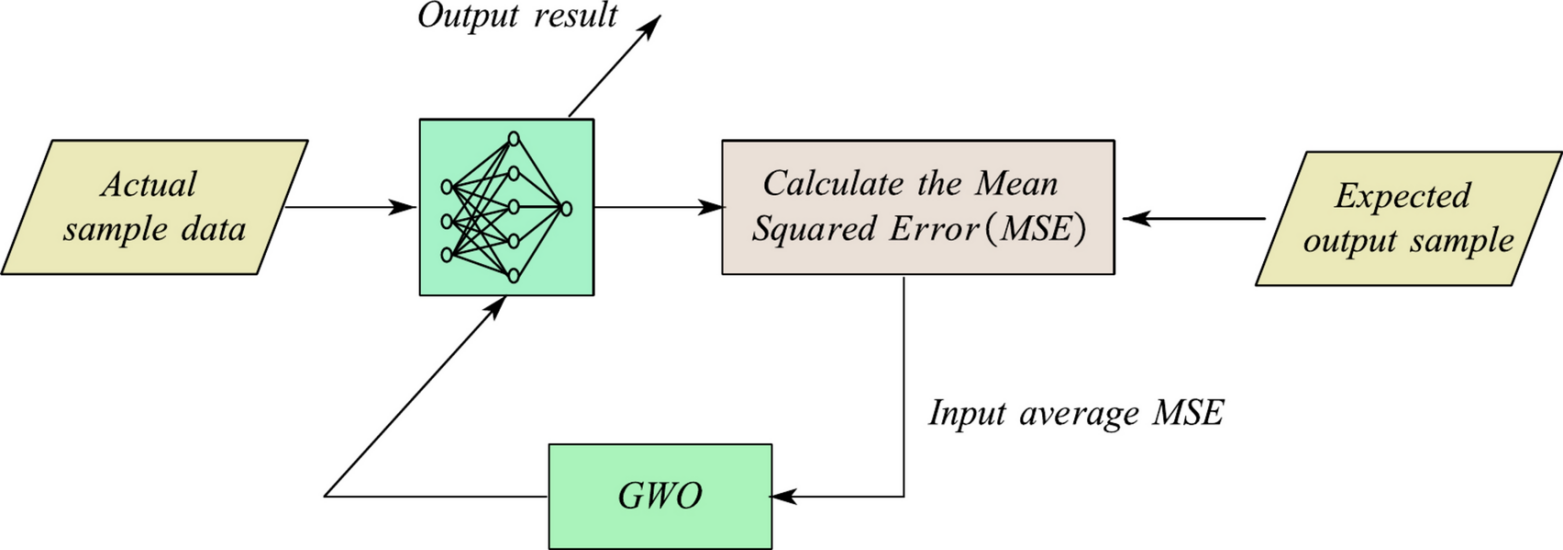
MLP based on GWO.

Figure 2 shows the schematic diagram of a multi-layer perceptron based on the CGWO algorithm. As shown in Fig. 2. The GWO algorithm iteratively trains by receiving the average MSE of all training samples and all expected output samples as the objective function. Through continuous iterative evolution, the connection weights and biases are adjusted to provide the optimal connection weights and biases for MLP after iterative training.

### User dynamic feature extraction module

#### Extraction of latent feature vectors for user ratings

In the user dynamic feature extraction module, the latent feature vector extraction of user behavior features includes the user behavior matrix input layer, feature vector layer, multi-layer MLP layer, and output layer. Assuming there are m users and n products, represented as $U = \{ u_{1} ,u_{2} , \ldots ,u_{n} \}$, and $P = \{ p_{1} ,p_{2} , \ldots ,p_{m} \}$, respectively. The user product behavior matrix is denoted as R, where $R_{ij}$ indicates the behavior record of user $u_{i}$ towards product $p_{j}$, such as clicking, browsing, or purchasing. When user $u_{i}$ has no behavior towards product $R_{ij} = 0$. The row data in the matrix represents the user’s behavior records for each product, while the column data represents the user behavior obtained by the product.

The user behavior feature matrix extracted through the behavior matrix is denoted as $U \in {\mathbf{R}}^{L \times n}$, while the product feature matrix is denoted as $P \in {\mathbf{R}}^{L \times m}$, where the vector $U_{i}$ represents the L-dimensional eigenvector of user $u_{i}$, and $P_{j}$ represents the L-dimensional eigenvector of product $p_{j}$. After obtaining the user behavior feature vector, it is input as input data into MLP. After deep learning of MLP, the potential feature vector $S_{u}$ of the user is finally obtained. The learning output results of each layer of MLP are represented as formula (8).

8 $$S_{u} = \sigma (R^{(k)} S^{(k - 1)} + b^{k} )$$

where s is the rating feature vector learned by user u through MLP, R is the i-th rating matrix, b is the i-th bias, k is the number of hidden layers in MLP, and $\sigma$ is the nonlinear activation function.

#### Extraction of latent feature vectors for user labeled tags

Usually, users label their favorite products or content on cross-border e-commerce platforms, and the label information can well reflect the user’s interests and preferences. Therefore, this article uses Convolutional Neural Networks (CNN) to extract user labeled label information and construct a user feature matrix. The process of extracting latent feature vectors for user annotated labels includes the label matrix input layer, embedding layer, user annotated label feature vector layer, pooling layer, and output layer.

Firstly, the sparse matrix vector of the labels is mapped to a dense matrix vector using word embedding techniques, and then a convolutional neural network is used for deep learning of this label information. Assuming there are k marketing labels, the label matrix annotated by users is denoted as $T_{ik} = \{ t_{1} ,t_{2} , \cdots ,t_{k} \}$, where $T_{ik}$ represents the set of labels that user $u_{i}$ annotated for product $t_{k}$. The user labels the product i as $T_{ui} = \{ t_{1} ,t_{2} , \cdots ,t_{k} \}$, where $t_{k}$ is the word embedding vector of the k-th text in the tag.

The embedding layer maps each word t in the label table to a $d_{t}$ dimensional vector, i.e. $t \in d_{t}$. If the same label appears repeatedly in multiple products, these labels share the same embedding representation. Convert each label into a word embedding matrix $T = \{ t_{1} ,t_{2} , \cdots ,t_{i + 1} , \cdots \} \in {\mathbf{R}}^{k \times L}$ using text embedding method, where L is the length of the label text.

In the cross-border intelligent marketing model, the convolutional layer in CNN is used to extract semantic feature vectors from user annotated label text. Assuming there are H convolutional filters, denoted as $F_{z} = \{ f_{1} ,f_{2} , \cdots ,f_{H} \}$. The input parameters of each filter are composed of a matrix $f_{z} \in {\mathbf{R}}^{l \times z}$, where l represents the size of the filter window and d represents the dimension of the embedding vector. The convolution calculation result of the z-th filter on the embedding matrix T can be expressed as:

9 $$C_{z} = ReLU(T_{c}^{z} *F_{z} + b_{z} )$$

where Relu represents the nonlinear activation function, $*$ represents the convolution operation, and $b_{z}$ is the deviation parameter of $T_{c}^{z}$. The label feature vector $C_{j} \in {\mathbf{R}}^{t - l + 1}$ generated by the jth filter is then used to obtain multiple features using the H convolution filter, as shown in Eq. (10)

10 $$C^{j} = (c_{1}^{j} ,c_{2}^{j} ,c_{3}^{j} , \ldots ,c_{t - l + 1}^{j} )$$

The main function of the pooling layer is to extract the maximum value from the feature vectors obtained through the convolutional layer. In this paper, the maximum pooling method is used to obtain the most important information, as shown in Eq. (11).

11 $$df = \max \left( {c_{1}^{j} ,c_{2}^{j} ,c_{3}^{j} , \ldots ,c_{t - l + 1}^{j} } \right)$$

Afterwards, it enters the fully connected layer, where the user rating feature vector and user label feature vector are weighted to obtain the latent feature vector representation of the user. The operation definition is shown in Eq. (12).

12 $$U_{i} = f(W \times df + R + b)$$

### Fusion module, predictive output module, and loss function

#### Fusion module, predictive output module

The user rating matrix is obtained through MLP learning, and the user annotation label matrix is obtained through convolutional neural network learning. The two are fused to obtain the user depth feature vector, and the fusion formula is shown in Eq. (13).

13 $$U_{i} = U_{Ri} + U_{{tag_{i} }}$$

Similarly, the item scoring matrix and item labeling matrix are obtained through perceptron learning and convolutional neural network calculation, respectively. The two are fused to obtain the item depth feature vector, and the fusion formula is shown in Eq. (14).

14 $$P_{j} = P_{Rj} + P_{{tag_{j} }}$$

Use Eq. (15) to fuse the user depth feature vector and the item depth feature vector to obtain the predicted value.

15 $$\hat{Y}_{ui} = \sigma (W^{T} (U_{i} \cdot P_{j} ))$$

where a is the activation function, which is used to better learn the nonlinear relationship between users and projects; B is a weight matrix used to learn the degree of connection between various vectors.

#### Network model of regularized loss function

Through feature extraction and feature sampling using MLP and pooling layers, the feature vectors generated in the fully connected layer are ultimately classified using the Softmax loss function of the output layer.

**A: Softmax loss function**

Logistic mainly targets binary classification problems, and the Softmax function improves logistic to solve multi classification problems. Assuming m input features $x^{(i)}$ and sample labels $y^{(i)}$ are denoted as

16 $$\{ (x^{(1)} ,y^{(1)} ),(x^{(1)} ,y^{(1)} ), \ldots ,(x^{(m)} ,y^{(m)} )\}$$

The loss function of Softmax regression is as follows

17 $$J(\theta ) = - \frac{1}{m}\left[ {\sum\limits_{i = 1}^{m} {\sum\limits_{j = 1}^{k} 1 } \left\{ {y^{(i)} = j} \right\}\ln (h_{\theta } (x^{(i)} ))} \right]$$

where

18 $$h_{\theta } (x^{(i)} ) = \frac{1}{{\sum\limits_{j = 1}^{k} {{\text{e}}^{{\theta_{j}^{T} x^{(i)} }} } }}\left[ {\begin{array}{*{20}c} {{\text{e}}^{{\theta_{1}^{T} x^{(i)} }} } \\ {{\text{e}}^{{\theta_{2}^{T} x^{(i)} }} } \\ \vdots \\ {{\text{e}}^{{\theta_{k}^{T} x^{(i)} }} } \\ \end{array} } \right]$$

where k is the number of types of sample labels, $\theta$ is the model parameter, and $h_{\theta } (x^{(i)} )$ is the hypothesis function used to predict the probability that sample $x^{(i)}$ belongs to each category. By minimizing the Softmax loss function, the value of $\theta$ can be obtained to estimate the category of a new sample. The essence of the Softmax loss function is to calculate all values to the power of e, sum them up, and then calculate the proportion of each value. Due to the mutual exclusion of classification results, the calculated label values are unique, ensuring that all samples in the dataset can only belong to one category.

**B: L2 norm regularization**

Small fluctuations in data often cause significant changes in the loss function values, leading to overfitting of the model and affecting predictive performance. The L2 norm can obtain smaller model parameter values by compressing regression coefficients, thereby avoiding overfitting. The L2 norm refers to the sum of the squares of all weights divided by the number of samples, and then multiplied by the regularization coefficient.

The Softmax loss function introducing L2 norm is as follows

19 $$H(\theta ) = J(\theta ) + \lambda_{1} ||\theta ||_{2}^{2}$$

where $||\theta ||_{2} = \sqrt {\sum\limits_{k = 1}^{k} {\sum\limits_{l = 1}^{l} {\theta_{kl}^{2} } } }$, $\lambda_{1}$ is an adjustable attenuation coefficient, which is also used as a training metric to ensure sparsity. L2 norm regularization can balance the reconstruction error term and the compression of regression coefficients. In this paper, $\lambda_{1} = 2$ is set. Regularization is the punishment of large numerical weights to enhance the model’s generalization ability. From the formula, it can be seen that the rule term a of L2 norm suppresses the weight of large values by imposing a square penalty on all parameters element by element. The extremely small value of a makes the weight very small, close to 0 but not equal to 0, ensuring a smoother coefficient vector. The output layer of MLP-GWO-CNN contains the L2 norm of ownership value coefficients. In this paper, we directly train and test the Softmax loss function by adding L2 norm regularization.

## Experimental analysis

This section presents the experimental results obtained from the proposed cross-border intelligent marketing system. By analyzing the results, the effectiveness of the system and its various components was evaluated. The experimental results have verified the performance of the system in the cross-border e-commerce environment, especially in user behavior modeling, personalized recommendation, and marketing strategy optimization, providing a more accurate and efficient solution for cross-border intelligent marketing.

### Experimental preparation

**Fig. 3:**
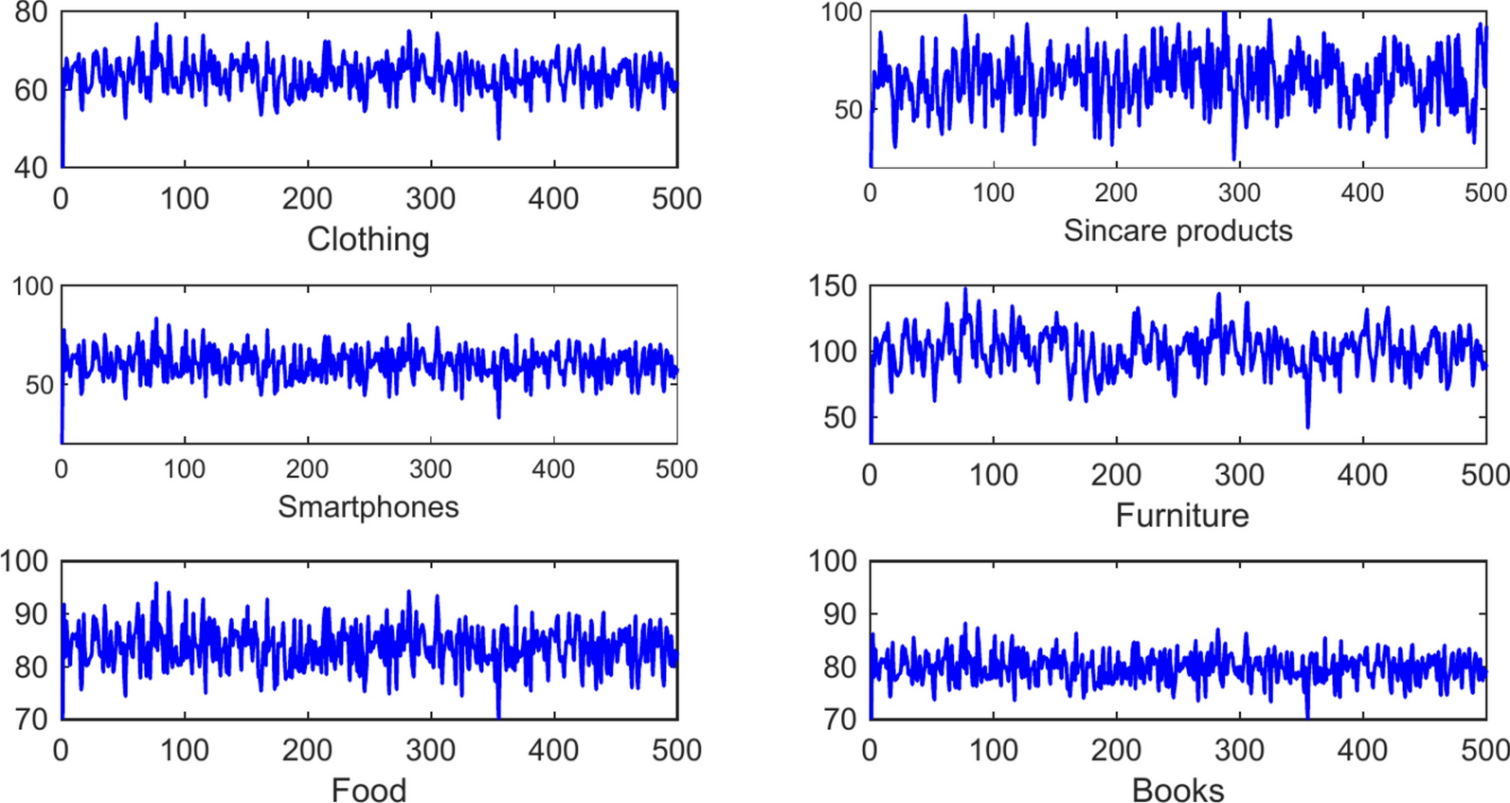
Different cross-border review datasets.

The application was written in Python 3.7 and the experiment was carried out within the PyCharm environment. The machine was set up with an Intel i7- 12700H processor, 16 GB of RAM, and a 64-bit version of Windows 11. This article collected a total of 12,953 data from September 2018 to September 2022 on the Amazon website^19^, including products such as Use Cross Border Clothing, Skincare Products, Smartphones, Furniture, Food, and Books. In the dataset, 80% are used as data machines and 20% are used as test sets. The description of different types of datasets is shown in the Fig. 3.

### Comparison algorithms

The experimental results of this article will be compared and analyzed with the following four recommendation algorithms to evaluate the performance of the proposed cross-border intelligent marketing system based on MLP-GWO-CNN:

RA1^20^ proposed a cross-border recommendation algorithm based on tensor Tucker decomposition and list level sorting learning. This algorithm is trained by optimizing the Mean Average Precision (MAP) to provide accurate recommendations based on user preferences. In this experiment, RA1 was used as one of the comparative algorithms.

DeepCoNN^21^: This model is based on a dual channel neural network, which learns user preferences from user reviews and extracts features of a product from all user reviews. The potential features of both are fused through layers at the top of the network to predict users’ ratings of products. This article uses DeepCoNN as a comparative algorithm to evaluate the text information extraction ability in cross-border marketing recommendations.

TTMF^22^: This is a matrix factorization recommendation model that combines label and temporal information. This model is modeled using a three-dimensional matrix of user rating data, item and tag information, and recommendation calculation is performed using gradient descent method. TTMF has advantages in integrating user tags and temporal dynamic information, and is therefore used to compare its time series processing capability with the algorithm proposed in this paper.

RA2^23^ proposed a novel recommendation method based on multi-source information fusion. This method calculates recommendation priority by combining user item rating data, user social network information, and item tags to achieve more personalized recommendations. RA2 is used to evaluate the recommendation effectiveness of the cross-border intelligent marketing system in this article under multidimensional information fusion.

### Experimental setup

Firstly, for the architecture and configuration of MLP components, this paper adopts a three-layer hidden layer structure, where the number of neurons in each layer is 128, 64, and 32 respectively. The activation function uses Leaky ReLU to enhance the model’s ability to express nonlinear features and alleviate the problem of gradient vanishing. The weight initialization adopts the He initialization strategy, the optimizer selects Adam, the initial learning rate is set to 0.001, and the dynamic adjustment mechanism (learning rate scheduler) is used to adjust according to the training error. In addition, the model is trained using a cross entropy loss function, with a maximum of 100 iterations set during the training process. The early stopping condition is that the validation set loss does not decrease for 10 consecutive epochs. To prevent overfitting, we added a 0.5 Dropout regularization after each layer.

Secondly, for the architecture of the CNN part, this article uses two convolutional networks and one fully connected layer. The kernel sizes of the convolutional layers are set to $3 \times 3$ and $5 \times 5$, with a stride of 1, and padding is used to preserve the size of the input features. Each convolutional layer is followed by a max pooling layer (pooling size $2 \times 2$) for downsampling to extract key features. In order to improve the expressive power of the model and prevent overfitting, we introduced Batch Normalization and Dropout operations after each convolution layer. The number of neurons in the fully connected layer is 128, and the activation function is also Leaky ReLU, ultimately outputting a 128 dimensional label embedding feature vector.

For the application of GWO in optimizing MLP training process, this paper adopts a matrix encoding strategy, which represents the weights and bias parameters of MLP as the search variables of GWO. In each iteration, GWO updates the position of the wolf pack based on the fitness values of individuals in the population (using the mean square error MSE of MLP as the fitness function), and dynamically adjusts the search step size to achieve a balance between global and local search. During training, the population size is set to 30 and the number of iterations is 50. The optimization objective is to minimize the error on the validation set. Through experiments, we found that the introduction of GWO significantly alleviates the sensitivity of MLP to initial values and the tendency to fall into local optima, while accelerating the convergence speed of the model.

Regarding the fusion of features from MLP and CNN, this paper proposes a fusion strategy based on weighted concatenation. Specifically, the feature vectors generated by MLP and CNN are first normalized to ensure consistent magnitudes of features from different sources. Then, a learnable weight matrix is used to weight and sum the two features, and a fully connected layer is added to the sum to further explore the interaction between features. The formula is expressed as:

20 $${\mathbf{F}}_{fusion} = \sigma \left( {{\mathbf{W}}_{mlp} \cdot {\mathbf{F}}_{mlp} + {\mathbf{W}}_{cnn} \cdot {\mathbf{F}}_{cnn} + {\mathbf{b}}} \right)$$

where ${\mathbf{F}}_{mlp}$ and ${\mathbf{F}}_{cnn}$ are the output eigenvectors of MLP and CNN respectively, ${\mathbf{W}}_{mlp}$ and ${\mathbf{W}}_{cnn}$ are learnable weighting matrices, b is the bias term, and $\sigma$ is the activation function.

### Experimental results

The performance indicators selected in this article include accuracy, recall rate, click through rate, recommendation rate, and user satisfaction. The experimental results are shown in the Tables 1, 2, 3 and 4.

**Table 1:** Accuracy under different methods (%).

	Clothing	Sincare products	Smartphones	Furniture	Food	Books
MLP-GWO-CNN	90.85	89.32	90.23	91.25	90.99	89.21
RA1	81.35	84.47	82.63	81.26	83.76	81.29
DeepCoNN	76.24	81.94	79.23	79.66	80.97	79.22
TTMF	78.39	79.36	78.26	77.38	80.12	80.67
RA2	77.66	77.29	80.26	80.93	81.83	82.96

**Table 2:** Recall rate under different methods (%).

	Clothing	Sincare products	Smartphones	Furniture	Food	Books
MLP-GWO-CNN	90.59	90.32	91.23	94.74	92.22	92.21
RA1	78.15	82.47	83.23	81.12	80.29	81.29
DeepCoNN	74.24	80.94	74.44	80.09	77.96	75.22
TTMF	76.32	76.35	85.56	81.29	81.28	81.67
RA2	78.62	72.19	81.28	82.16	81.96	81.96

**Table 3:** Click through rate under different methods (%).

	Clothing	Sincare products	Smartphones	Furniture	Food	Books
MLP-GWO-CNN	92.85	91.34	91.63	92.71	91.22	91.89
RA1	82.35	82.27	81.63	82.72	82.63	80.97
DeepCoNN	79.24	82.99	79.64	81.09	80.96	82.73
TTMF	73.39	78.16	80.96	82.19	80.28	79.12
RA2	76.61	78.23	81.98	80.86	77.42	81.13

**Table 4:** Recommendation rate under different methods (%).

	Clothing	Sincare products	Smartphones	Furniture	Food	Books
MLP-GWO-CNN	96.23	95.12	96.31	94.96	95.19	94.87
RA1	86.26	79.19	82.49	79.69	82.73	86.67
DeepCoNN	84.89	84.49	83.56	84.81	84.92	82.73
TTMF	86.93	82.26	84.18	80.93	85.61	82.15
RA2	81.86	81.97	82.13	77.61	81.97	83.91

It can be observed that the method proposed in this article achieves high performance across various evaluation metrics. The accuracy, recall rate, click-through rate, and recommendation rate surpass 89%, 90%, 91%, and 94%, respectively. These results demonstrate the effectiveness of the proposed approach in providing accurate and relevant recommendations to users.

Regarding the statistical significance of model performance, this article conducted multiple experiments and p-value tests to ensure that the reported performance indicators are not accidental. This article uses t-test and analysis of variance to compare the differences between the proposed model (MLP-GWO-CNN) and other comparative algorithms (RA1, DeepCoNN, TTMF, RA2) in multiple evaluation metrics. The experimental results show that the proposed model has significant statistical advantages over other benchmark algorithms in terms of accuracy, recall, click through rate, and recommendation rate, with p-values less than 0.01, indicating that these performance improvements are statistically significant.

**Fig. 4:**
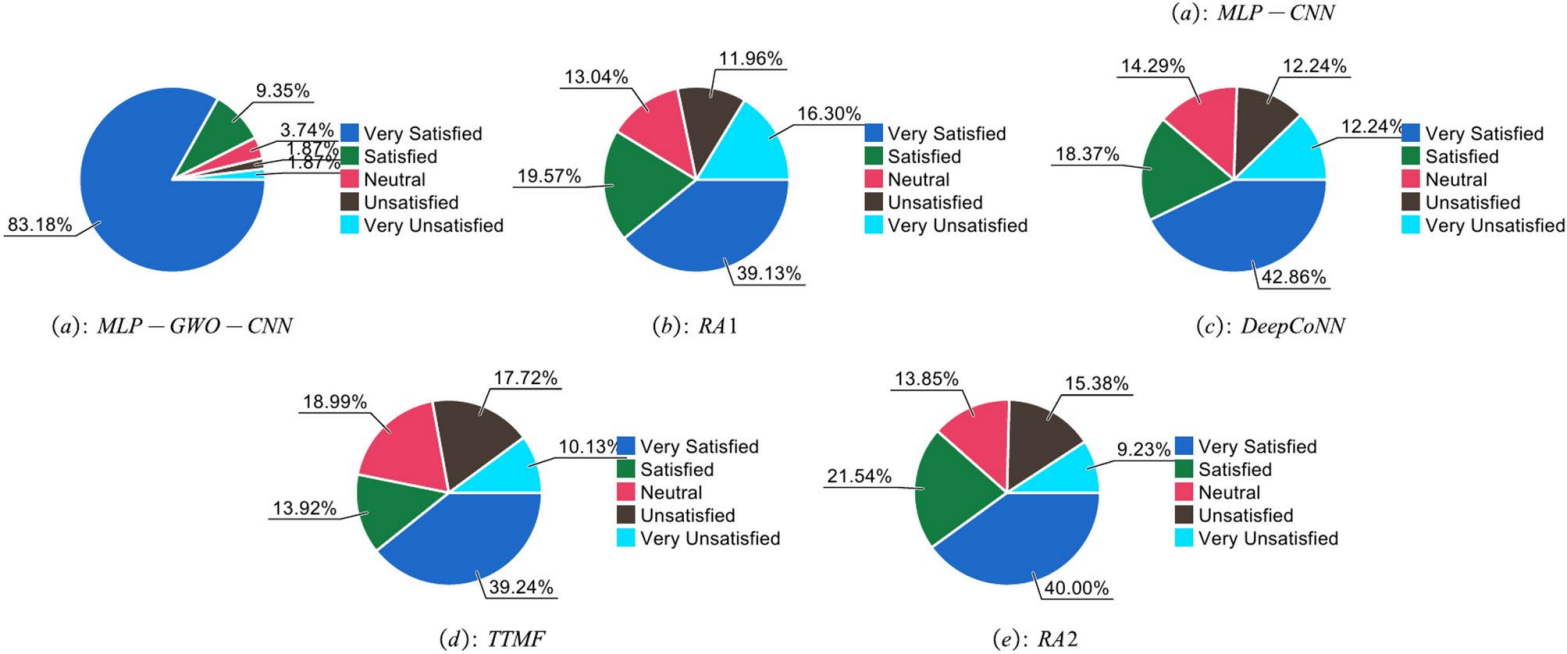
Proportion of user satisfaction under different methods.

Figure 4 shows the customer satisfaction of various strategies. After multiple tests, the user satisfaction of traditional push systems ranged from 0.2 to 0.5, while the push system proposed in this article achieved a user satisfaction rate of over 0.96. This indicates that compared to standard marketing push systems, the cross-border intelligent marketing push system proposed in this article has higher user satisfaction.

**Fig. 5:**
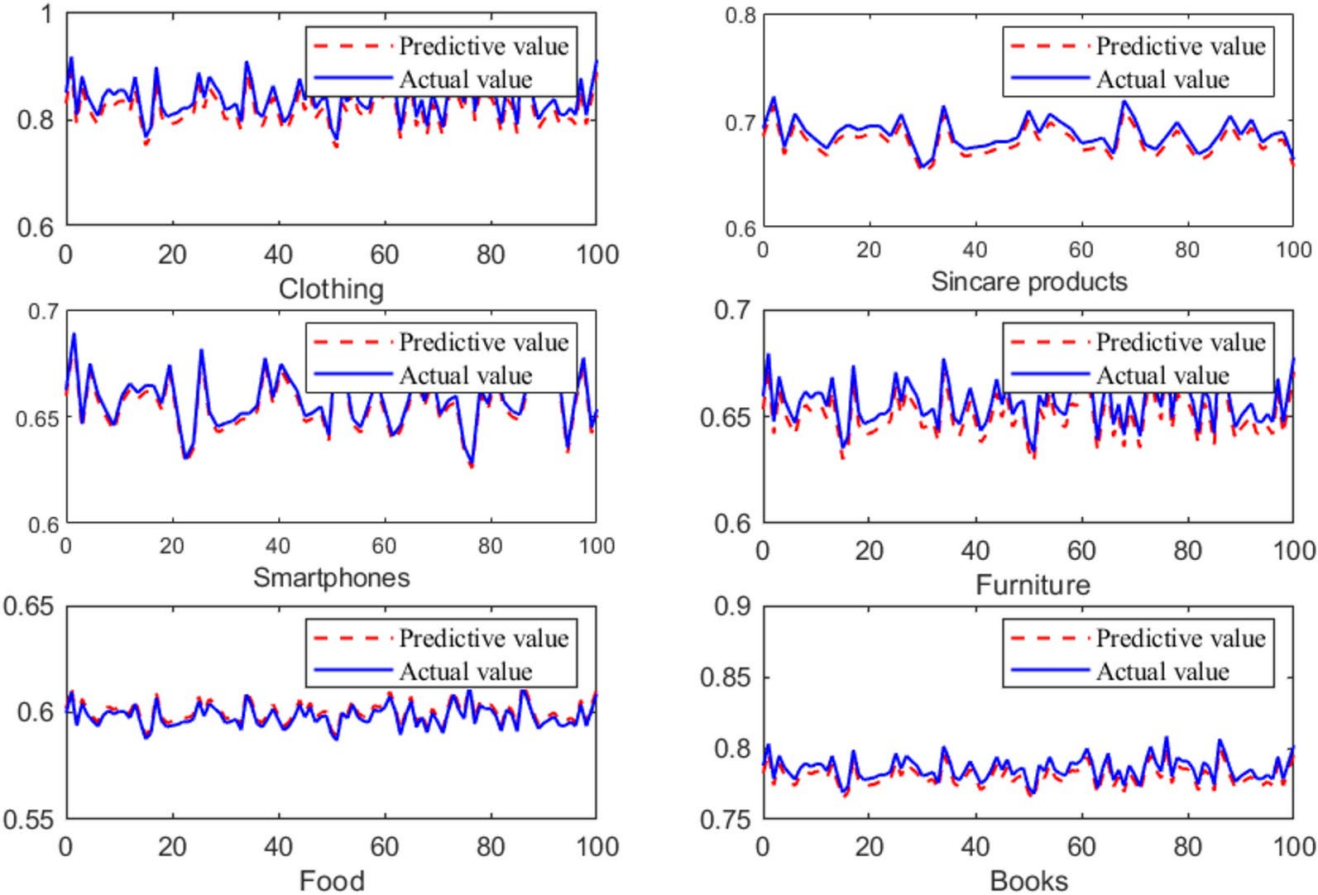
The prediction results of the cross border intelligent marketing system developed in this article.

This article conducted prediction experiments on different products, and the experimental results are shown in the Fig. 5. It can be seen that the cross border intelligent marketing developed in this article has similar predicted and actual results for related car accessories.

To verify the effectiveness of the proposed method, this paper introduces data from eBay^25^ and AliExpress^26^ platforms, covering different types of product categories and user behavior patterns. On the eBay dataset, we found that by combining MLP and CNN to extract user behavior and label features, the recommendation accuracy for low-frequency products can be significantly improved. On the AliExpress dataset, GWO optimization further improved the training efficiency and accuracy of the model in high-dimensional data.

In the sparse dataset experiment, we used user behavior data from different cross-border e-commerce platforms, which contained a large number of users’ partial ratings or no rating records of products, showing significant sparsity. To verify the robustness of the model in this situation, we compared traditional collaborative filtering methods and matrix factorization model recommendation methods to evaluate their performance in handling sparse data. In the experiment, we first performed missing value imputation on the dataset and trained on this basis. The experimental results are shown in Table 5. Our method outperforms other benchmark models significantly in accuracy, recall, and Normalized Discounted Cumulative Gain (NDCG), especially in recommendations for low-frequency users and cold start users, demonstrating strong robustness. By introducing the GWO optimization algorithm, we further optimized the training process of the model on sparse data, reducing its sensitivity to initial weights and local optima, thereby effectively improving the accuracy and stability of recommendations.

**Table 5:** Performance comparison of different models under sparse data).

	Accuracy	Recall	NDCG
MLP-GWO-CNN	83.74	79.24	80.54
Collaborative filtering model	60.15	44.20	61.78
Matrix factorization model	57.90	42.89	58.18

### Ablation experiments

**Fig. 6:**
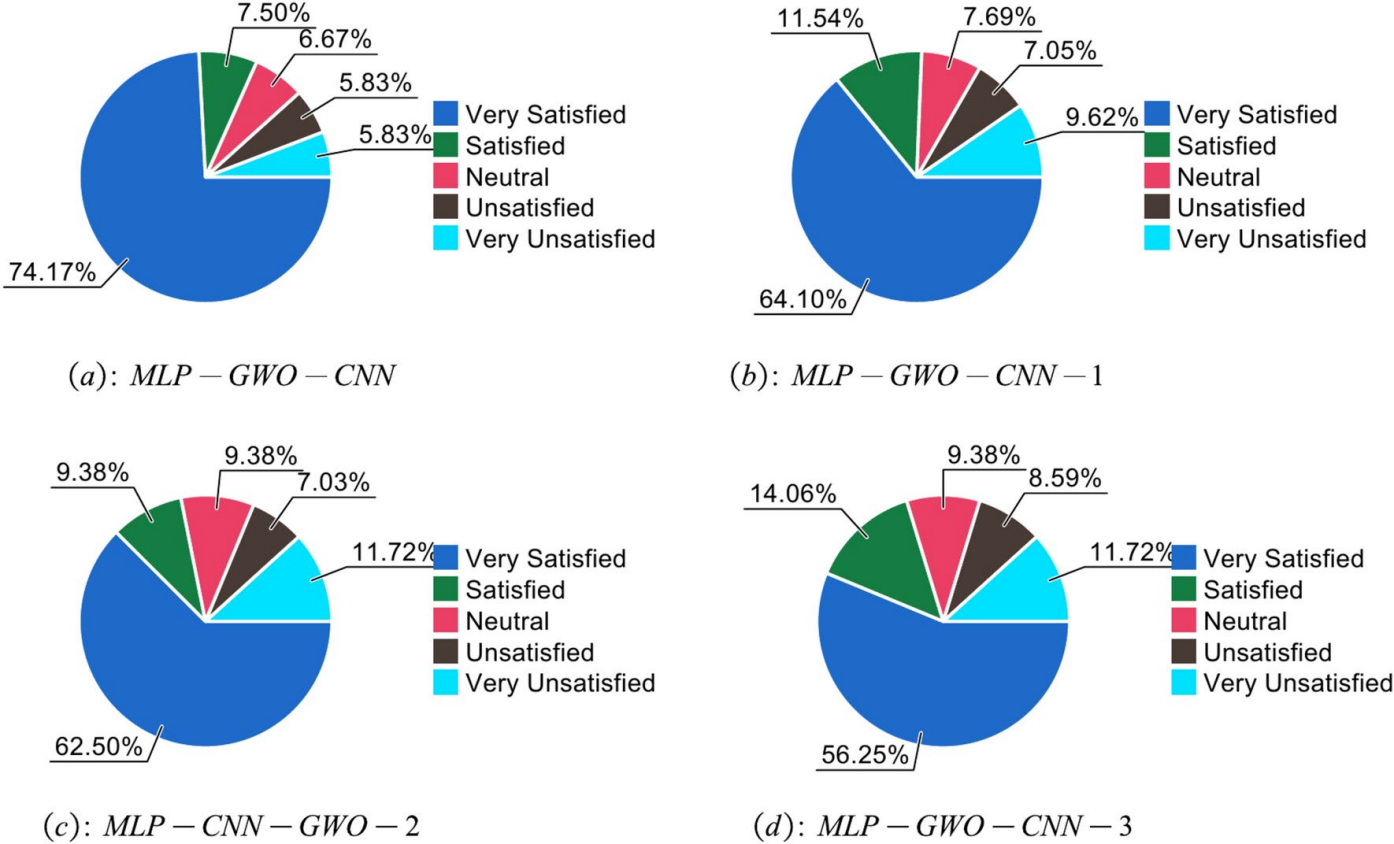
Proportion of user satisfaction under MLP-GWO-CNN, MLP-GWO-CNN-1, MLP-GWO-CNN-2, MLP-GWO-CNN-3.

In order to verify the effectiveness of the MLP-GWO-CNN algorithm designed in this paper, ablation experiments were conducted. The method without MLP is called MLP-GWO-CNN-1, the method without social labels is called MLP-GWO-CNN-2, and the method without item scoring is called MLP-GWO-CNN-3. The experimental results are shown in Fig. 6 and Table 6.

**Table 6:** Results of ablation experiment (%).

	Accuracy	Recall rate	Click through rate	Recommendation rate
MLP-GWO-CNN	92.63	93.13	91.97	95.18
MLP-GWO-CNN-1	81.96	84.20	82.71	83.71
MLP-GWO-CNN-2	86.23	81.72	80.97	84.59
MLP-GWO-CNN-3	82.92	80.97	81.86	83.91

It can be seen that the addition of MLP, social tags, and project ratings significantly improves user satisfaction, further demonstrating the effectiveness of the cross-border intelligent marketing system developed in this article.

In order to evaluate the contribution of each component separately, we have added four variant models based on the original ablation experiment: (1) using only MLP without combining CNN features (referred to as MLP--only); (2) Using only CNN without incorporating MLP features (referred to as CNN--only); (3) Excluding GWO optimization, only randomly initialized MLP (referred to as No--GWO) was used, and the experimental results are shown in the Table 7.

**Table 7:** Results of ablation experiment (%).

	Accuracy	Recall rate	Click through rate	Recommendation rate
MLP-GWO-CNN	92.63	93.13	91.97	95.18
MLP-only	82.96	80.78	85.12	86.10
CNN-only	79.29	82.15	86.41	86.10
No-GWO	84.01	83.18	84.86	79.48

In comparison, it was found that when using MLP or CNN alone, the accuracy and recall of the model significantly decreased, indicating that the complementarity between the two in feature extraction is crucial for improving performance. In addition, after removing GWO optimization, the training time of the model significantly increased, while the convergence accuracy decreased, verifying the key role of GWO in improving the robustness and optimization efficiency of MLP.

### Error analysis

This article categorizes and analyzes the prediction errors of the model, mainly including false positives and false negatives. In terms of false positives, the model is prone to misjudgment on some popular products or products with universal labels, especially when user behavior data is sparse or label information is incomplete. This type of error is common in product category boundary areas, such as when a user is interested in a certain type of product but does not match in dimensions such as price and brand. In the future, we plan to improve the recommendation performance of these high-risk categories by introducing more refined labels or reinforcement learning. In terms of false negatives, the model sometimes fails to identify potential products of interest, especially when the user’s historical behavior is sparse or there is limited label information. New users or low-frequency buyers often fail to recommend products of interest. To address this issue, we will optimize the cold start problem by incorporating social tags, product reviews, and other information to supplement user profiles and reduce false negative errors.

## Discussion

Although MLP and CNN each have their own advantages in recommendation systems, with the former being adept at processing numerical rating data and the latter being suitable for processing spatially structured label information, typically used to extract historical rating and social label features separately, there are few studies that combine the two, especially in complex data environments such as cross-border e-commerce. Our innovation lies in designing a dual path network structure that utilizes MLP and CNN to extract user and product features from different dimensions, and achieves comprehensive user interest modeling through a feature fusion layer. Through this fusion, the model can simultaneously utilize historical rating and social tag data to more accurately capture users’ diverse needs, thereby improving the effectiveness of personalized recommendations.

In addition, the introduction of GWO is also our innovation. Traditional MLP is susceptible to the influence of initial weights during training and is prone to getting stuck in local optima. Although deep learning has powerful expressive power, traditional optimization methods struggle to find global optima in complex data and high-dimensional features. To overcome this problem, we adopted the GWO optimization algorithm, which simulates the process of gray wolf hunting for global search, especially in high-dimensional sparse data and nonlinear problems, with significant advantages. This enables GWO to effectively enhance the global search capability and training efficiency of MLP, thereby improving the overall performance of the recommendation system.

The experimental results show that when using MLP, CNN, or GWO alone, the model performance is not as good as the composite model we proposed. When dealing with cold start issues and sparse data, using MLP alone can result in lower accuracy and recall, while CNN performs poorly in digitizing historical data. By combining MLP and CNN, we are able to more comprehensively explore user behavior and social label features, improving recommendation accuracy. After introducing GWO, the training process becomes more efficient, avoiding local optima and further improving model performance.

In reference^24^, the author proposed the Evolutionary Convergence Algorithm (ECA), which has strong global search capability and can avoid local optimal problems. However, the reason for choosing GWO instead of ECA is as follows: although ECA performs well in some optimization problems, its algorithm complexity is relatively high, especially when dealing with large-scale data and high-dimensional features, with high computational overhead. In contrast, the GWO optimization algorithm achieves global search by simulating the hunting behavior of grey wolves, which has lower computational complexity and can effectively avoid local optima problems. It also demonstrates good performance in cross-border e-commerce recommendation systems. In addition, based on practical application experience, GWO has advantages in sparse data and nonlinear model optimization.

## Conclusions

This article proposes a cross-border intelligent marketing system design based on multi-layer perceptron convolutional neural network, aiming to address the shortcomings of traditional marketing methods in the context of big data and personalized needs. By introducing a combination model of Multi Layer Perceptron-Grey Wolf Optimization (MLP-GWO) and Convolutional Neural Network (CNN), the system proposed in this paper can not only effectively extract deep level latent information from user behavior, social labels, and product features, but also maintain high recommendation accuracy when facing sparse and complex cross-border e-commerce data. The experimental results show that the system outperforms existing mainstream algorithms in key indicators such as recommendation accuracy, recall, click through rate, and recommendation rate, further verifying the applicability of the model in cross-border marketing scenarios.

However, cross-border intelligent marketing systems need to face users from different cultural and linguistic backgrounds. Therefore, future research will further explore natural language processing (NLP) technology, especially improvements in multilingual processing and multicultural adaptability, so that the system can better understand and adapt to the preferences and needs of users in different countries and regions, and enhance the user experience in the global market. In future work, we plan to further strengthen the consideration of cultural and behavioral diversity to improve the universality of recommendation systems in different regions and cultural backgrounds.

## Data Availability

The dataset can be accessed upon request. Corresponding author can be contacted for queries or data needs.
